# Luteinizing hormone induces ovulation via tumor necrosis factor α-dependent increases in prostaglandin F_2α_ in a nonmammalian vertebrate

**DOI:** 10.1038/srep14210

**Published:** 2015-09-16

**Authors:** Diego Crespo, Frederick W. Goetz, Josep V. Planas

**Affiliations:** 1 Departament de Fisiologia i Immunologia, Facultat de Biologia, Universitat de Barcelona and Institut de Biomedicina de la Universitat de Barcelona (IBUB), Avinguda Diagonal 643, 08028 Barcelona, Spain; 2 School of Freshwater Sciences, University of Wisconsin-Milwaukee, 600 E. Greenfield Avenue, WI 53204, USA

## Abstract

Ovulation is induced by the preovulatory surge of luteinizing hormone (LH) that acts on the ovary and triggers the rupture of the preovulatory ovarian follicle by stimulating proteolysis and apoptosis in the follicle wall, causing the release of the mature oocyte. The pro-inflammatory cytokine tumor necrosis factor α (TNFα) and prostaglandin (PG) F_2α_ (PGF_2α_) are involved in the control of ovulation but their role mediating the pro-ovulatory actions of LH is not well established. Here we show that Lh induces PGF_2α_ synthesis through its stimulation of Tnfα production in trout, a primitive teleost fish. Recombinant trout Tnfα (rTnfα) and PGF_2α_ recapitulate the stimulatory *in vitro* effects of salmon Lh (sLh) on contraction, proteolysis and loss of cell viability in the preovulatory follicle wall and, finally, ovulation. Furthermore, all pro-ovulatory actions of sLh are blocked by inhibition of Tnfα secretion or PG synthesis and all actions of rTnfα are blocked by PG synthesis inhibitors. Therefore, we provide evidence that the Tnfα–dependent increase in PGF_2α_ production is necessary for the pro-ovulatory actions of Lh. The results from this study shed light onto the mechanisms underlying the pro-ovulatory actions of LH in vertebrates and may prove important in clinical assessments of female infertility.

Ovulation is a complex process leading to the release of the mature oocyte from the ovarian follicle and is induced by the surge of luteinizing hormone (LH)^1^. The important role of LH in ovulation is demonstrated by the inability of LH receptor null mice to ovulate^2^. Part of the necessary events for LH-induced ovulation include the weakening of the follicle wall by proteolytic digestion, apoptotic follicle cell death and follicle contraction. Overall, these coordinated events are crucial for follicle rupture and subsequent expulsion of the oocyte in mammals. The LH preovulatory surge stimulates the ovarian production of key factors involved in ovulation such as members of the matrix metalloproteinase (MMP) system and tissue inhibitors of MMPs (TIMPs) that are involved in the regulation of the gonadotropin-induced ovarian follicle wall degradation and breakdown in mammals^3^. The MMP family includes MMP2 (also named gelatinase A), known to regulate the dynamics of the ovarian extracellular environment prior to ovulation by digesting collagen^3^. In fact, MMP2 expression is stimulated in response to LH^4^ and its collagenolytic activity increases before ovulation^5^. Furthermore, proteolytic enzymes belonging to other families, including plasminogen activators/plasmin and a disintegrin and metalloproteinase with thrombospondin-like motifs (ADAMTS), have important roles in the remodeling of the extracellular matrix (ECM) during ovulation^3^. In addition to the increase in proteolytic activity, apoptotic cell death contributes to the weakening of the ovarian follicle wall and facilitates its localized degradation^6^.

In mammals, there is evidence suggesting that prostaglandins (PGs) and the pro-inflammatory cytokine tumor necrosis factor α (TNFα) participate in regulating key aspects of the ovulatory process. However, their precise involvement in mediating the stimulatory effects of LH on ovulation has not been established to date. PGs are known to induce apoptosis in the mammalian ovary^7^ and to stimulate collagenolytic activity during ovulation^8^. The levels of PGs F_2α_ (PGF_2α_) and E_2_ (PGE_2_) in follicular fluid peak just before ovulation^1^ and ovulation is blocked by the PG synthesis inhibitor indomethacin (INDO), which has led to the suggestion that INDO inhibits follicular rupture by preventing the preovulatory increase in ovarian PG synthase activity^9^. The preovulatory LH surge induces the expression of PG G/H synthase 2 (PTGS2)^10^ and LH stimulates PGF_2α_ and PGE_2_ production by rat preovulatory follicles *in vitro*^11^. Further evidence for the involvement of PGs in ovulation is derived from the fact that mice deficient in PTGS2 fail to ovulate^12^. On the other hand, TNFα is also secreted by mammalian preovulatory follicles^13^ and ovarian and serum TNFα levels in rats are increased by *in vivo* administration of hCG^14^. TNFα enhances ovulation rates in rat ovary perfusates^15^ and, like PGs, stimulates apoptosis^16^ and collagenolytic activity in preovulatory follicles^17^. Interestingly, biosynthesis of PGs in rat preovulatory follicles is stimulated by TNFα^18^. These findings clearly suggest that both PGs and TNFα are possible mediators of the pro-ovulatory effects of LH but whether they are part of the same cascade of events triggered by LH to stimulate ovulation has not been directly shown.

Like in mammals, LH is also indispensible for ovulation in teleost fish, a group of primitive vertebrates. This is shown by the ability of fish Lh to stimulate ovulation *in vitro* in medaka (*Oryzias latipes*) and brook trout (*Salvelinus fontinalis*)^19^,^20^ and by the requirement of a functional Lh and a Lh receptor for successful ovulation in zebrafish (*Danio rerio*)^21^,^22^. Also like in mammals, several enzymes belonging to different proteolytic systems have been shown to be involved in ovulation in teleost fish. Initial studies reported an increase at the time of ovulation in the activity of collagenolytic metalloproteases in brook trout and yellow perch (*Perca flavescens*)^23^ and in the abundance of a serine protease similar to kallikrein (KT14)^24^ as well as serine protease inhibitors (trout ovulatory proteins, TOPs)^25^, believed to protect the ovary from non-specific degradation by proteases produced at the time of ovulation, in brook trout. More recently, elegant studies in medaka have shown that follicle rupture involves the initial degradation of laminin, a component of the follicle basement membrane, by the serine protease plasmin, that is activated by plasminogen activator (Plau1) which in turn is under the control of the plasminogen activator inhibitor-1 (Pai1)^26^,^27^. These proteolytic events are believed to be followed in time by activation of MT-MMP1 (also named Mmp14), MT-MMP2 (also named Mmp15) and Mmp2, the enzyme responsible for the degradation of collagen IV, the second component of the basement membrane, and that is under the control of the tissue inhibitor of matrix metalloproteinase-2b (Timp2b)^28^, leading to the complete rupture of the follicle during ovulation. In fish, evidence is emerging to suggest that the major initial endocrine signal regulating these proteolytic systems during ovulation in teleosts is Lh in view of the stimulatory effects of piscine Lh on the ovarian expression of different members of the MMP family^19^,^20^, including Mmp2, as well as Pai1^27^. Furthermore, several lines of evidence also suggest an important regulatory action of PGs and members of the TNF family during the preovulatory period in teleosts. Importantly, PGF_2α_ has been identified as the most effective inducer of *in vitro* ovulation in certain teleost species^29^,^30^ and Tnfα has been shown to be involved in the weakening of the follicle wall by stimulating follicle contraction and granulosa cell apoptosis in preovulatory brown trout (*Salmo trutta*) follicles^31^. In the present study, we show that the stimulation of ovulation by Lh in trout requires a Tnfα–dependent increase in the production of PGF_2α_, likely one of the final effectors of the pro-ovulatory events orchestrated by Lh.

## Results

### sLh stimulates contraction and proteolytic activity through the production of Tnfα

**Figure 1:**
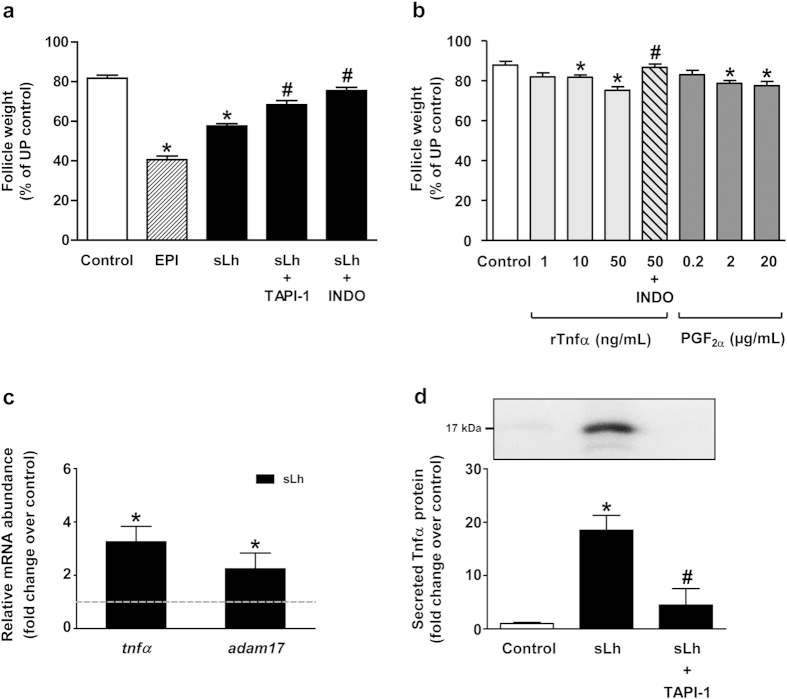
Mediatory effects of Tnfα and PGF_2α_ on sLh-induced contraction of brown trout preovulatory follicles. (**a**) Effects of sLh on follicle contraction. Punctured follicles were incubated for 16 h at 15 °C with epinephrine (EPI; 10 μM), sLh (25 ng/mL), sLh plus TAPI-1 (50 μM) and sLh plus INDO (10 μg/mL). The results are expressed as percent change with respect to the unpunctured control group that was set at 100%. (**b**) Effects of rTnfα and PGF_2α_ on follicle contraction. Punctured preovulatory follicles were incubated for 16 h at 15 °C with rTnfα (1, 10 and 50 ng/mL), rTnfα (50 ng/mL) in the presence of INDO (10 μg/mL) and PGF_2α_ (0.2, 2 and 20 μg/mL). In (**a**) and (**b**), data are expressed as percent change with respect to the unpunctured control group that was set at 100%. (**c**) Relative expression of *tnfα* and *adam17* analyzed by qPCR in ovarian follicles incubated in the absence or presence of sLh (25 ng/mL). (**d**) Ovarian Tnfα secretion in brown trout follicle incubates by Western blot. Preovulatory follicles were incubated with sLh (25 ng/mL) in the absence or presence of TAPI-1 (50 μM) for 16 h at 15 °C. A representative Western blot is shown in the inset and the full-length blot is presented in Supplementary Figure S1. In all graphs, data are the mean ± standard error (SEM) (n = 3–5). *P < 0.05, with respect to control; ^#^*P* < 0.05, with respect to sLh (**a,d**) and with respect to rTnfα (50 ng/mL) (**b**).

*In vitro* exposure of isolated brown trout (*Salmo trutta*) preovulatory follicles to sLh, as well as to the positive control epinephrine (EPI)^32^, significantly stimulated follicle contraction, shown by the decrease in follicle weight in punctured follicles when compared to controls (Fig. 1a). In view of the known mediation of the maturational effects of sLh by Tnfα and the direct stimulatory effects of recombinant trout Tnfα (rTnfα) on follicle contraction and granulosa cell apoptosis in trout preovulatory follicles^31^,^33^, we initially investigated the possible involvement of Tnfα in mediating the pro-ovulatory effects of sLh. First, TNFα-processing inhibitor-1 (TAPI-1), an effective inhibitor of Tnfα secretion in trout^33^, blocked sLh-stimulated follicle contraction (Fig. 1a). Second, rTnfα directly stimulated follicle contraction in a dose-dependent manner (Fig. 1b). Third, sLh stimulated *tnfα* mRNA levels (Fig. 1c) and Tnfα secretion that was blocked by TAPI-1 (Fig. 1d; Supplementary Figure S1). Finally, sLh stimulated the mRNA levels of *adam17* (Fig. 1c), the protease Tnfα-converting enzyme that is a target of TAPI-1, and of a tumor necrosis factor receptor (Table 1). These data suggest that the stimulatory effects of sLh on follicle contraction are mediated by Tnfα.

**Figure 2:**
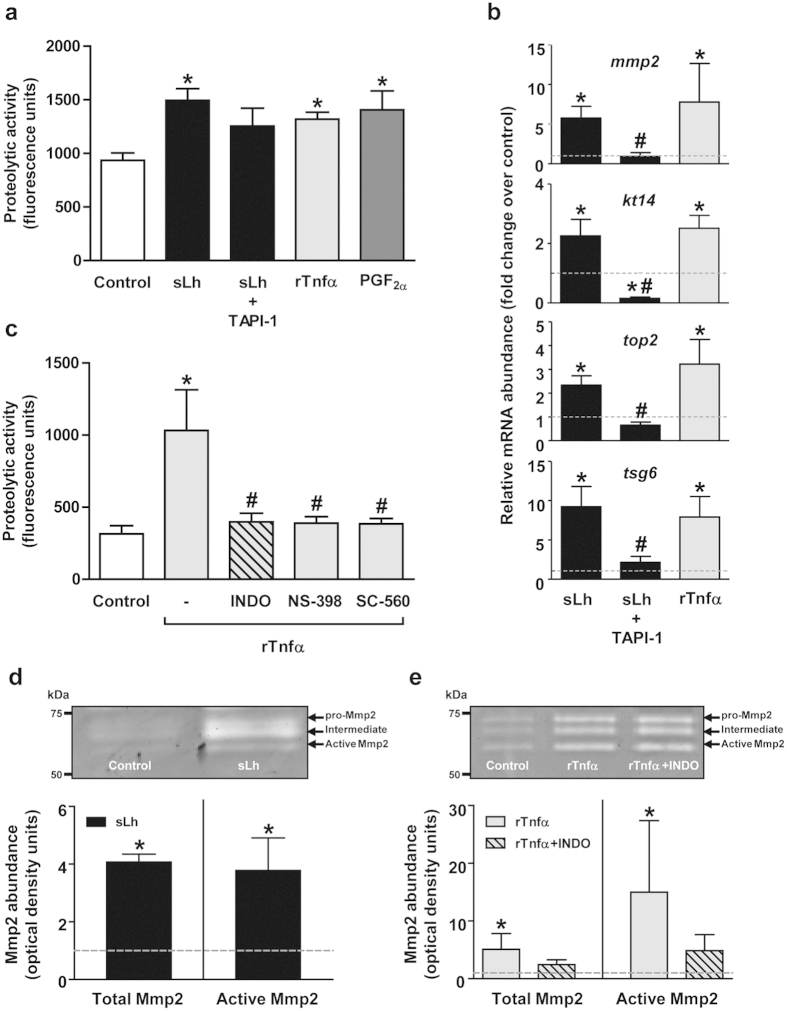
Induction of proteolysis by Lh, Tnfα and PGF_2α_ in brown trout preovulatory follicles. (**a**) *In vitro* effects of sLh (25 ng/mL), in the absence or presence of TAPI-1 (50 μM), rTnfα (50 ng/mL) and PGF_2α_ (2 μg/mL) on trout gelatinase/collagenase activity in ovarian follicle homogenates. (**b**) Expression of proteolytic genes in ovarian follicles incubated with sLh (25 ng/mL), in the absence or presence of TAPI-1 (50 μM), or with rTnfα (50 ng/mL). The relative expression of *mmp2*, *kt14*, *top2* and *tsg6* was determined by qPCR and normalized to the abundance of *18s*. (**c**) Inhibition of rTnfα-induced gelatinase/collagenase activity in ovarian follicle homogenates by prostaglandin synthesis inhibitors. Follicles were incubated with rTnfα (50 ng/mL) in the absence or presence of INDO (10 μg/mL), NS-398 (10 μg/mL) and SC-560 (10 μg/mL) for 16 h at 15 °C. (**d,e**) Determination of ovarian Mmp2 activity by gelatin zymography in follicles treated with sLh (25 ng/mL) (**d**) and rTnfα (50 ng/mL) in the absence or presence of INDO (10 μg/mL) (**e**). Clear bands in the zymogram indicated enzymatic digestion of gelatin. Densitometric analyses of Mmp2 gelatinase activity were quantified and expressed as fold increase of the total (pro-, intermediate and active Mmp2) or active Mmp2 content in control (set at 1 and represented by the dashed line) and sLh and rTnfα ± INDO-treated follicles. Representative zymograms are shown in the graph and the full-length gels are presented in Supplementary Figure S3. In all graphs, data are the mean ± SEM (n = 3–4). *P < 0.05, with respect to control; ^#^*P* < 0.05, with respect to sLh (**b**) and with respect to rTnfα (50 ng/mL) (**c**).

We next investigated whether sLh regulates proteolytic activity in brown trout preovulatory follicles. Our results show that sLh significantly increased collagenolytic activity in intact follicles (Fig. 2a) and the mRNA expression levels of genes known to participate in the weakening of the follicle wall in fish, such as *mmp2*, *kt14* and *top2*^24^,^28^,^34^, in intact follicles (Fig. 2b) and in isolated theca layers (Supplementary Figure S2). In isolated granulosa layers, sLh also increased the mRNA levels of *mmp2* and *kt14* but *top2* was not expressed (Supplementary Figure S2). Microarray analysis of intact follicles incubated with sLh evidenced enrichment of functional classes and pathways associated with cell communication and ECM remodeling, among others (Supplementary Table S1). Differentially expressed genes involved in proteolysis included serine protease 23 (*prss23*), ADAM metallopeptidase with thrombospondin type 1 (*adamts1*), cathepsin L (*ctsl*), *mmp23b*, *mmp19*, plasminogen activator inhibitor-1 (*pai1*) and *mmp2*, all up-regulated, and metalloproteinase inhibitor 2 (*timp2*) and *mmp9*, that were down-regulated (Table 1 and Supplementary Table S2). sLh also induced a general increase in the expression of ECM constituents such as collagen type I and IV, fibronectin and laminin (Table 1). Gelatin zymography of brown trout preovulatory follicles revealed the presence of proteinases with a molecular weight corresponding to that of pro- and active Mmp2 and showed that sLh increased significantly the amount of total and active Mmp2 protein (Fig. 2d; Supplementary Figure S3). Abrogation of gelatinolytic activity in brown trout preovulatory follicles by broad MMP inhibitors (EDTA, batimastat and 1, 10-phenanthroline) and by MMP2-specific inhibitors (MMP-2 inhibitor IV) and the similar electrophoretic size of gelatinolytic activity of recombinant human MMP2 and trout ovarian samples support the notion that the observed gelatinolytic activity is likely attributable to Mmp2 (Supplementary Figure S4; data not shown). The effects of sLh on proteolysis appeared to be mediated by Tnfα because rTnfα, like sLh, stimulated proteolytic activity (Fig. 2a,c), the mRNA expression levels of the proteolytic genes *mmp2*, *kt14* and *top2* (Fig. 2b), albeit only in theca layers (Supplementary Figure S2), and the amount of total and active Mmp2 protein (Fig. 2e). In support of a mediatory role for Tnfα in the pro-ovulatory actions of Lh, TAPI-1 blocked the stimulatory effects of sLh on proteolytic activity (Fig. 2a) and the mRNA expression levels of *mmp2*, *kt14* and *top2* in intact follicles (Fig. 2b) and isolated theca layers (Supplementary Figure S2). In isolated granulosa layers, *kt14* was the only proteolytic gene whose expression was significantly stimulated by rTnfα, although TAPI-1 blocked the stimulatory effects of sLh on *mmp2* and *kt14* mRNA levels (Supplementary Figure S2). Interestingly, sLh also stimulated the expression of TNFα-stimulated gene 6 (*tsg6*), important for the formation of the cumulus ECM and fertility of the mammalian oocyte^1^, in intact follicles (Fig. 2b) and isolated theca and granulosa layers (Supplementary Figure S2). rTnfα directly stimulated *tsg6* mRNA expression in intact follicles (Fig. 2b), although exclusively in theca layers (Supplementary Figure S2), and TAPI-1 significantly blocked the effects of sLh (Fig. 2b, Supplementary Figure S2). Therefore, these results suggest that sLh stimulates proteolysis in the follicle wall of brown trout preovulatory follicles and that its effects are mediated by Tnfα.

### PGF_2α_ mediates the stimulatory effects of sLh and rTnfα on contraction and proteolytic activity

**Figure 3:**
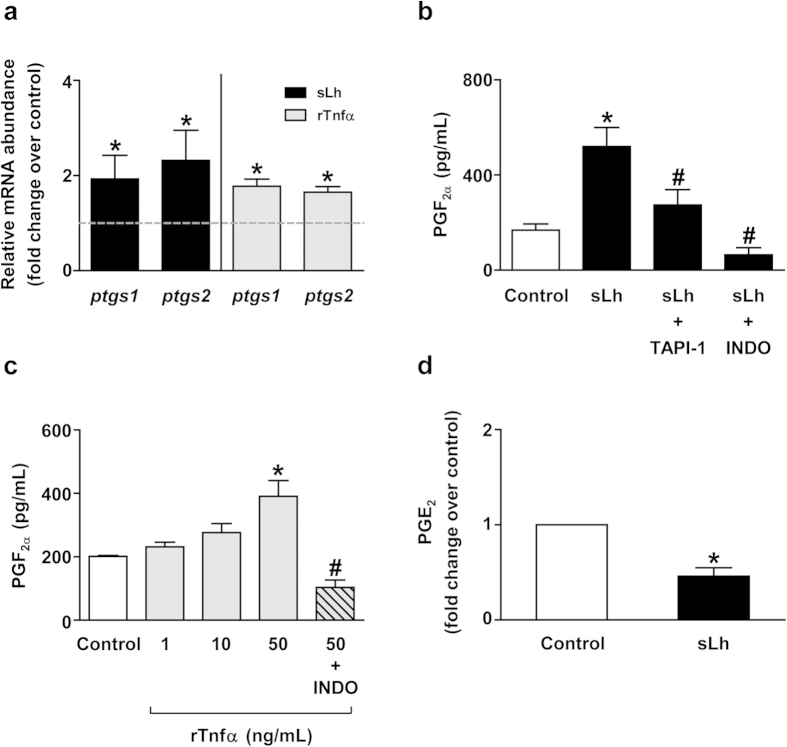
Regulation of prostaglandin synthesis by Lh and Tnfα in brown trout preovulatory follicles. (**a**) *In vitro* effects of sLh (25 ng/mL) and rTnfα (50 ng/mL) on the expression of enzymes involved in prostaglandin synthesis. The relative expression of *ptgs1* and *ptgs2* was determined by qPCR and normalized to the abundance of *18s*. (**b**) Determination of PGF_2α_ production *in vitro* by intact ovarian follicles treated with sLh. Trout follicles were incubated with sLh (25 ng/mL) in the absence or presence of TAPI-1 (50 μM) and INDO (10 μg/mL) for 16 h at 15 °C and PGF_2α_ was measured in the incubation media. (**c**) PGF_2α_ production in response to rTnfα treatment *in vitro*. Follicles were incubated with rTnfα (1, 10 and 50 ng/mL) or rTnfα (50 ng/mL) with INDO (10 μg/ml) for 16 h at 15 °C. (**d**) PGE_2_ production by brown trout preovulatory ovarian follicles. Follicles were incubated in the absence or presence of sLh (25 ng/mL) for 16 h at 15 °C and PGE_2_ levels were measured in the incubation media. In all graphs, data are the mean ± SEM (n = 3–7). *P < 0.05, with respect to control; ^#^*P* < 0.05, with respect to sLh (**b**) and with respect to rTnfα (50 ng/mL) (**c**).

Next, we asked whether the effects of sLh and rTnfα on contraction and proteolysis of brown trout preovulatory follicles are mediated, in turn, by PGF_2α_, a known stimulator of contraction and ovulation of trout preovulatory follicles^29^,^35^. Inhibition of PG synthesis with INDO significantly blocked sLh- and rTnfα-induced follicle contraction while PGF_2α_ alone stimulated follicle contraction, as previously shown^35^, mimicking the effects of sLh and rTnfα (Fig. 1a,b). Furthermore, INDO as well as SC-560 and NS-398, two selective inhibitors of PTGS1 and PTGS2, respectively, significantly blocked the stimulatory effects of rTnfα on proteolytic activity (Fig. 2c) and INDO significantly blocked rTnfα-stimulated increase of total and active Mmp2 protein abundance (Fig. 2e). The involvement of PGF_2α_ as a mediator of the effects of sLh and rTnfα was further suggested, first, by the stimulation of *ptgs1* and *ptgs2* mRNA levels by sLh and rTnfα (Fig. 3a); second, by the stimulation of PGF_2α_ production by sLh and rTnfα (Fig. 3b,c); third, by the inhibition of the stimulatory effects of sLh on PGF_2α_ production by TAPI-1 and INDO (Fig. 3b) and, finally, by the inhibition of rTnfα-stimulated PGF_2α_ production by INDO (Fig. 3c). In contrast to the stimulation of PGF_2α_ production by sLh, the production of PGE_2_, reported to block ovulation in trout^29^, was inhibited by sLh (Fig. 3d). These results suggest that PGF_2α_ mediates the stimulatory effects of Lh and Tnfα on follicle contraction and proteolytic activity in brown trout preovulatory follicles.

### sLh, rTnfα and PGF_2α_ induce the loss of viability of follicular cells

**Figure 4:**
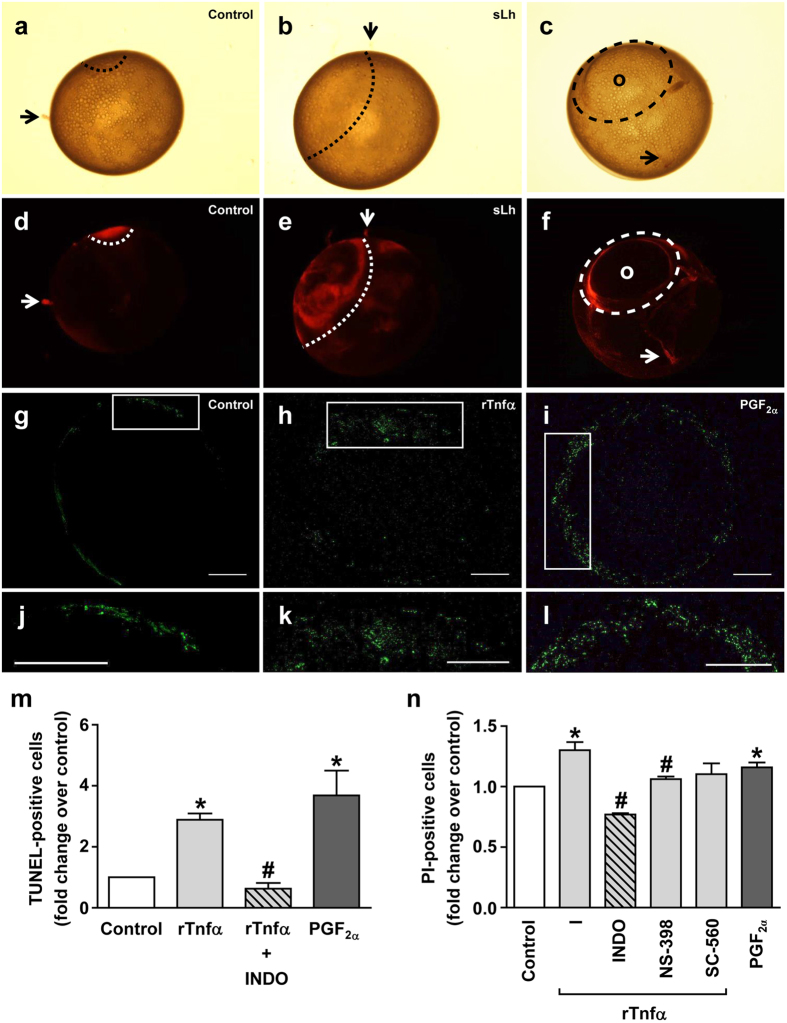
Lh, Tnfα and PGF_2α_ induce cell death in trout preovulatory follicles. (**a–f**) Assessment of cell death in ovarian follicles. Cell viability was assessed in follicles incubated for 18 h at 12 **°**C in the absence (**a,d**) or the presence of sLh (25 ng/mL) (**b,e**) and subsequently follicles were stained with propidium iodide (PI; 20 μg/mL) during 10 min at room temperature and visualized under bright light (**a,b**) or a fluorescent stereomicroscope (**d,e**). An ovulating follicle incubated under control conditions showing the point of rupture (**c,f**) (o, oocyte). Loss of cell viability is highlighted by a dashed line and stromal tissue debris is indicated by arrows. Representative pictures of a total of three independent experiments are shown. (**g–l**) Representative images of preovulatory follicles subjected to TUNEL analysis. Follicles incubated in the absence (control; **g**,**j**) or presence of rTnfα (50 ng/mL; **h**,**k**) and PGF_2α_ (2 μg/mL; **i**,**l**) were analyzed by TUNEL. Green staining indicates fragmented DNA in cells undergoing apoptosis. Scale bar represents 1000 μm. (**m**) Quantification of TUNEL-positive cells. Quantification was performed using ImageJ software. (**n**) Analysis of apoptosis in isolated granulosa cells by PI staining. Granulosa cells isolated from trout preovulatory follicles incubated in the absence or presence of rTnfα (50 ng/mL), rTnfα plus PG synthesis inhibitors (INDO, NS-398 and SC-560; 10 μg/mL) and PGF_2α_ (2 μg/mL) were stained with PI and analyzed by flow cytometry (FACS analysis). Data are the mean ± SEM (n = 3). *P < 0.05, with respect to control; ^#^*P* < 0.05, with respect to rTnfα.

sLh decreased cell viability in the follicle layer of preovulatory brook trout follicles as shown by propidium iodide (PI) staining, revealing the loss of cell viability in a localized area in the follicle that likely represented the site of follicle rupture, when compared to control follicles (Fig. 4a,b,d,e). In brook trout follicles undergoing ovulation, cell death was clearly observed as a ring immediately surrounding the site of rupture and the protruding oocyte (Fig. 4d,f). Having shown that Tnfα and PGF_2α_ may be important mediators in the regulation of follicle contraction and proteolysis by sLh prior to ovulation, we next investigated whether rTnfα and PGF_2α_ could induce the loss of viability of brown trout follicle cells. First, we observed the presence of a few number of apoptotic cells in control preovulatory follicles that were mainly localized in a small region of the follicle, as assessed by TUNEL assay (Fig. 4g,j). Incubation with rTnfα caused a significant, localized increase in the number of apoptotic cells in brown trout preovulatory follicles and INDO blocked this effect (Fig. 4h,k,m). In support of the possible PG involvement in follicle cell apoptosis, PGF_2α_ strongly increased the number of TUNEL-positive cells (Fig. 4m) and their distribution appeared to take place across the entire surface of the follicle (Fig. 4i,l). Second, analysis of apoptosis in granulosa cells by PI staining and flow cytometry demonstrated that rTnfα significantly stimulated apoptosis and that INDO and NS-398, but not SC-560, significantly blocked this effect (Fig. 4n). Finally, incubation with PGF_2α_ significantly increased the number of PI-positive granulosa cells (Fig. 4n). Consistent with the sLh-induced loss of viability in follicle wall cells, sLh affected the expression of genes involved in the regulation of apoptosis, as assessed by microarray analysis (Table 1). The expression of pro-apoptotic genes such as growth arrest and DNA-damage-inducible protein gadd45 beta ii, involved in TNFα-induced apoptosis, forkhead box O3A (also called FKHRL1; an important factor that promotes apoptosis in mammals) and Bcl2 antagonist of cell death (a pro-apoptotic Bcl2 family member) were significantly up-regulated by sLh. However, the expression of a small set of pro-apoptotic genes was also down-regulated in response to sLh (e.g. cell death activator CIDE-3, death-associated protein kinase3, programmed cell death protein 2). Therefore, our results suggest that sLh induces cell death by apoptosis in trout follicle cells through the local action of Tnfα and PGF_2α_.

### sLh-induced ovulation requires the mediatory effects of rTnfα and PGF_2α_

**Figure 5:**
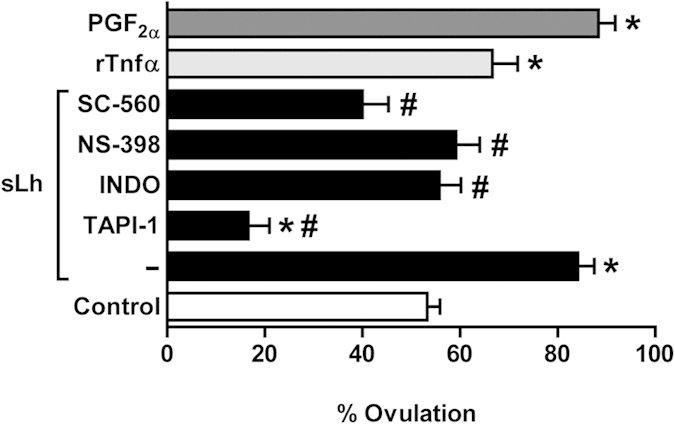
Effects of Lh, Tnfα and PGF_2α_ on *in vitro* ovulation in brook trout preovulatory follicles. Trout follicles were incubated for 36 h at 12 °C in the presence or absence of sLh (25 ng**/**mL), sLh plus different inhibitors (TAPI-1, 50 μM; INDO, 10 μg/mL; NS-398, 10 μg/mL; and SC-560, 10 μg/mL), rTnfα (50 ng/mL) and PGF_2α_ (2 μg/mL). Data are the mean ± SEM (n = 4). *P < 0.05, with respect to control; ^#^*P* < 0.05, with respect to sLh.

We investigated the direct effects of sLh, rTnfα and PGF_2α_ on *in vitro* ovulation using preovulatory follicles from brook trout, an appropriate salmonid model species for studying *in vitro* ovulation^20^,^29^. As previously shown^20^, sLh significantly stimulated *in vitro* ovulation in brook trout follicles (Fig. 5). Importantly, rTnfα and PGF_2α_ also significantly stimulated *in vitro* ovulation (Fig. 5). TAPI-1 completely blocked sLh-induced *in vitro* ovulation, supporting a role for Tnfα in mediating the pro-ovulatory effects of sLh. Interestingly, the percentage of ovulated follicles in the presence of sLh and TAPI-1 was even lower than that observed in the control group and appeared to be related to the ability of TAPI-1 to block spontaneous (in the absence of sLh) ovulation (Supplementary Figure S5). Furthermore, INDO, NS-398 and SC-560 significantly blocked the stimulatory effect of sLh on *in vitro* ovulation (Fig. 5), strongly suggesting that PGF_2α_ may be involved in sLh-induced ovulation in trout. Like TAPI-1, INDO and SC-560, but not NS-398, were able to inhibit significantly *in vitro* spontaneous ovulation (Supplementary Figure S5). Validation of the involvement of PGF_2α_ in mediating the effects of sLh in brook trout preovulatory follicles was shown by the ability of sLh to significantly stimulate the mRNA expression levels of *ptgs1* and *ptgs2* and the production of PGF_2α_ (Supplementary Figure S6). As in brown trout, sLh also inhibited the production of PGE_2_ (Supplementary Figure S6M). These results strongly suggest that Tnfα and PGF_2α_ mediate the pro-ovulatory effects of sLh in trout.

## Discussion

In this study, we show that Lh induces ovulation in trout by stimulating proteolysis, cell death and contraction of the preovulatory follicle wall through the intraovarian production of Tnfα and PGF_2α_. Although these two mediators were previously postulated to be independently involved in the control of mammalian ovulation^1^,^15^, this study strongly suggests that Lh orchestrates the ovulatory response by promoting a Tnfα-dependent increase in the production of PGF_2α_, likely one of the final mediators of the pro-ovulatory actions of Lh in the vertebrate ovary.

Here we show that sLh directly stimulates the secretion of Tnfα by trout preovulatory follicles by stimulating the expression of *tnfα* and the expression and activity of *adam17*, an enzyme that cleaves the integral transmembrane precursor Tnfα protein into the mature, soluble secreted form of Tnfα, and that is the target of the inhibitor TAPI-1. These results are consistent with the reported stimulation of ovarian and serum TNFα levels by *in vivo* administration of hCG in rats^14^. As previously shown in immature preovulatory trout ovarian follicles^33^, Tnfα is exclusively produced by the theca layer, similar to mammalian preovulatory follicles^36^. Furthermore, we also show that sLh and Tnfα stimulate the production of PGF_2α_, as in mammals^11^,^18^, as well as the expression of the PG synthesis enzymes *ptgs1* and *ptgs2* in trout preovulatory follicles. These results, coupled with the inability of sLh to stimulate PGF_2α_ synthesis when Tnfα secretion is blocked, strongy suggest that sLh stimulates the production of PGF_2α_ in a Tnfα-dependent manner.

In agreement with the notion that TNFα is an important factor for follicle rupture and ovulation in mammals^3^,^36^, our study provides evidence to suggest that Lh-induced ovarian Tnfα stimulates *in vitro* ovulation in vertebrates. This conclusion is supported by our observations on the ability of rTnfα to stimulate *in vitro* ovulation independently of Lh and by the complete block of sLh-induced ovulation when follicular sLh-stimulated Tnfα secretion is inhibited by TAPI-1. Our results complement previous studies demonstrating that TNFα enhances ovulation rates elicited by LH in rats^15^ and that ovulation is blocked by the intrafollicular injection of TNFα antiserum in ewes^16^. The pro-ovulatory actions of Tnfα in the trout preovulatory follicle involve stimulation of proteolytic activity, apoptosis and follicle contraction and remarkably recapitulate the observed pro-ovulatory actions of Lh. These include the stimulation of proteolytic activity through the induction of Mmp2, both at the level of mRNA expression and content of total and active protein, by both rTnfα and sLh. The observation that in mammals TNFα stimulates collagenolytic activity^17^ and the mRNA expression of MMP2^5^, suggests that the proteolytic action of TNFα is well conserved in vertebrates. In medaka, Mmp2 is believed to degrade collagen type IV^28^, the main constituent of the basement membrane that separates the theca and granulosa cell layers, although *mmp2* expression is not regulated by Lh in this species^19^. Based on these observations, we suggest that Tnfα mediates sLh-induced proteolytic activity in trout preovulatory follicles at least in part through its stimulation of Mmp2 activity, contributing to the degradation of the basement membrane and to the weakening of the ECM in the follicle wall. Interestingly, comparison of the transcriptomic profile of trout preovulatory follicles stimulated with sLh (this study) and rTnfα^31^ evidences the induction of similar proteolytic enzyme systems (e.g. MMPs, plasminogen/plasmin and ADAMTS) reported to be important for ovulation in mammals^3^ and in fish^37^. In addition, rTnfα, like sLh, causes cell death in the follicle layer of trout preovulatory follicles, specifically by inducing apoptosis in granulosa cells, as previously shown in this species^31^ and in mammals^16^, further contributing to the weakening and eventual rupture of the follicle wall prior to ovulation. Therefore, we propose that Lh-induced ovarian Tnfα has the dual role of promoting collagen breakdown and cellular death in the trout preovulatory follicle as a requisite for ovulation.

As in mammals, PGs are known inducers of ovulation in fish^37^,^38^. Specifically, in trout, PGF_2α_ directly stimulates *in vitro* ovulation^29^,^39^ and follicle contraction^35^ and its role in inducing ovulation is supported by the reported increase of PGF_2α_ plasma levels during ovulation^40^. However, the precise actions and the regulation of the production of PGF_2α_ are not fully understood, even in mammals. In the present study, we show that PGF_2α_ mimics the pro-ovulatory effects of Lh and Tnfα in trout as evidenced by its ability to stimulate proteolysis, cell death and contraction of the follicle wall as well as ovulation independently of Lh and Tnfα. Having shown that Tnfα mediates the pro-ovulatory effects of Lh, the inhibition of the stimulatory effects of rTnfα on proteolysis, both at the level of proteolytic activity and amount of total and active Mmp2, on apoptosis and on the contraction of the trout preovulatory follicle wall by INDO and/or PTGS1- and PTGS2-specific inhibitors, strongly suggest that PGF_2α_ is a mediator of Lh-induced ovarian Tnfα. The identification, regulation and spatial localization of the PGF_2α_ receptor in relation to the formation of the rupture site in trout preovulatory follicles are aspects worthy of investigation in future studies.

In summary, we provide evidence strongly supporting that Lh orchestrates the complex events leading to ovulation involving the stimulation of proteolysis, apoptosis and contraction of the follicle wall through the stimulation of the production of PGF_2α_ in a Tnfα-dependent process (Supplementary Figure S7). The results from this study contribute to our understanding of the precise mechanisms by which LH induces ovulation in vertebrates and may also provide clues as to the nature of pathological states involving alterations in LH signaling in the human ovary.

## Methods

### Animals

Reproductively mature female brown and brook trout that had naturally undergone germinal vesicle breakdown (GVBD; i.e. oocyte maturation) but not yet ovulated were staged and sampled as previously described^20^. The experimental protocols used for brown and brook trout in this study have been reviewed, approved and carried out in accordance with the Ethics and Animal Welfare Committee of the University of Barcelona, Spain and by the Institutional Animal Care and Use Committee of the University of Wisconsin-Milwaukee, respectively.

### Ovarian tissue incubations

Trout preovulatory (post-GVBD) ovaries were isolated and incubated as previously described^33^. Theca and granulosa layers were manually dissected from ovarian follicles after treatment with the test compounds, as previously described, with the theca layer having up to a 10% contamination with granulosa cells^41^. Purified sLh from coho salmon (*Oncorhynchus kisutch*) pituitaries was a kind gift from Dr. Penny Swanson (Northwest Fisheries Science Center, NOAA Fisheries, Seattle, WA, USA)^42^. rTnfα, shown to be biologically active in the trout ovary^31^,^33^, was produced as previously described^43^. In all experiments performed in this study, preovulatory follicles were incubated in Hank’s balanced salt solution containing 0.2% bovine serum albumin (Sigma-Aldrich, fraction V) in 6 well plates (10 follicles/4 mL/well; in triplicate) in the absence or presence of the test compounds under shaking conditions (100 rpm). Follicles were cultured at 12 °C (brook trout) or 15 °C (brown trout), temperatures selected to match the different conditions that the two species were raised in. PGF_2α_, EPI, TAPI-1 and PG synthesis inhibitors were purchased from Cayman Chemical Company, Sigma-Aldrich or Calbiochem. Follicle contraction and ovulation rate were determined as previously described^20^,^31^. PGF_2α_ and PGE_2_ were analyzed directly in the incubation media using commercial immunoassays (Cayman).

### *In vitro* gelatinase/collagenase activity

Brown trout follicles incubated in the absence or presence of the test compounds were homogenized in 100 mM Tris, 200 mM NaCl, 0.1% Triton X-100 (pH 7.4) and centrifuged at 10,000 × *g* for 20 min at 4 °C. The total gelatinase/collagenase activity in the supernatants was determined by the EnzCkek Gelatinase/Collagenase assay Kit (Molecular Probes). The reaction mix was incubated at room temperature in the dark and fluorescence was determined after 24 h and expressed as average relative fluorescence units of activity (the background signal was subtracted).

### Immunoblotting and gelatin zymography

Immunoblots of supernatants of brown trout ovarian follicle incubates using a rabbit polyclonal antibody against rTnfα^43^ were performed as previously described^33^. For zymography, ovarian follicles were homogenized in 1× Zymogram Development Buffer (BioRad) containing 1% Triton X-100 and gelatinases were purified using gelatin sepharose 4B (GE Healthcare) as previously described^44^. Purified samples were loaded onto 10% SDS-polyacrylamide gels containing 0.1% gelatin (Panreac). After electrophoresis, enzymes were renatured by washing with three changes of 2.5% Triton X-100 in distilled H_2_O for 30 min at room temperature followed by a 42 h incubation at 37 °C in 1 × Zymogram Development Buffer under gentle agitation. To demonstrate that the gelatinolytic activity could be due to MMP2, incubations were performed in the absence or presence of broad MMP inhibitors (20 mM EDTA; 10 μM batimastat, Sigma-Aldrich; 1 mM 1,10-phenanthroline, Sigma-Aldrich) and a specific MMP2 inhibitor (10 μM MMP-2 Inhibitor IV, Calbiochem). Gels were stained with Coomassie blue (0.1% in 30% methanol, 10% glacial acetic acid and 60% distilled H_2_O) and destained (30% methanol, 10% glacial acetic acid and 60% distilled H_2_O) for 1 h. Clear bands in the zymogram indicated enzymatic digestion of gelatin. Densitometric analysis of Mmp gelatinase activity was quantified by ImageJ software.

### Gene expression analyses

RNA extraction and qPCR was performed as previously described^33^. Gene expression values by qPCR were expressed as fold change relative to control and normalized for each gene against those obtained for *18s*. Primer sequences, GenBank accession numbers or ProbeID (genes included in the microarray platform used in this study) of the target genes and PCR product sizes are shown in Supplementary Table S3. Microarray analyses were performed with RNA samples from control and sLh-treated preovulatory follicles from four separate brown trout females (n = 4) that were labeled with Cy3 and Cy5 using the Two-Color Low Input Quick Amp Labeling kit (Agilent Technologies) following a dye swap experimental design. Labeled cRNAs were hybridized to an Agilent custom trout oligonucleotide (4 × 44 K) microarray (GEO GPL16819), annotated with the STARS bioinformatics package, as previously validated and described^45^. Differentially expressed genes were selected according to fold change > 1.8 and *P* < 0.01 (sample *t*-test). The data discussed in this publication have been deposited in NCBI’s Gene Expression Omnibus and are accessible through GEO Series accession number GSE67592 (http://www.ncbi.nlm.nih.gov/geo/query/acc.cgi?acc = GSE67592). Twelve genes were validated by qPCR (Supplementary Table S2).

### Analysis of follicle cell apoptosis

Cell viability was assessed in intact brook trout preovulatory follicles that were incubated in PBS and exposed to propidium iodide (PI; Sigma-Aldrich) staining buffer (20 μg/mL PI, 0.1% Triton X-100) for 10 min at room temperature. Follicles were briefly washed with PBS after the incubation period and visualized using an Olympus SZX12 fluorescent stereomicroscope. To determine the incidence of apoptosis, frozen ovarian follicles that were previously incubated in the absence or presence of the test compounds were subjected to deoxynucleotidyl transferase-mediated dUTP nick-end labeling (TUNEL). First, follicles were washed with PBS (pH 7.4) and subsequently fixed in 4% PBS-buffered paraformaldehyde for 20 min at room temperature. After a 30 min wash with PBS, follicles were treated with permeabilization solution (0.1% Triton X-100, 0.1% sodium citrate) for 5 min on ice. Finally, follicles were incubated with TUNEL reaction mixture (*In Situ* Cell Death Detection Kit, Fluorescein; Roche) in the dark at 37 °C for 1 h. After washing twice in PBS, follicles were mounted in Fluoromount antifade mounting reagent (Sigma-Aldrich) and visualized under a fluorescence stereomicroscope (Olympus MVX10 MacroView). Quantification of TUNEL-positive cells was performed using the ImageJ software. Apoptosis in isolated granulosa cells was performed by PI staining and FACS analysis as previously described^31^.

### Statistical analyses

Statistical differences were calculated by the non-parametric Kruskal-Wallis test followed by Mann-Whitney U test or one-way analysis of variance (ANOVA) followed by the Fisher protected least significant different test for the determination of differences among groups, using StatView 5.0 (SAS Institute, Cary, NC, USA). Results are expressed as mean ± SEM and differences between groups were considered to be significant if *P* < 0.05.

## Additional Information

**How to cite this article**: Crespo, D. *et al.* Luteinizing hormone induces ovulation via tumor necrosis factor α-dependent increases in prostaglandin F_2α_ in a nonmammalian vertebrate. *Sci. Rep.* **5**, 14210; doi: 10.1038/srep14210 (2015).

## Supplementary Material

Supplementary Information

## Figures and Tables

**Table 1:**
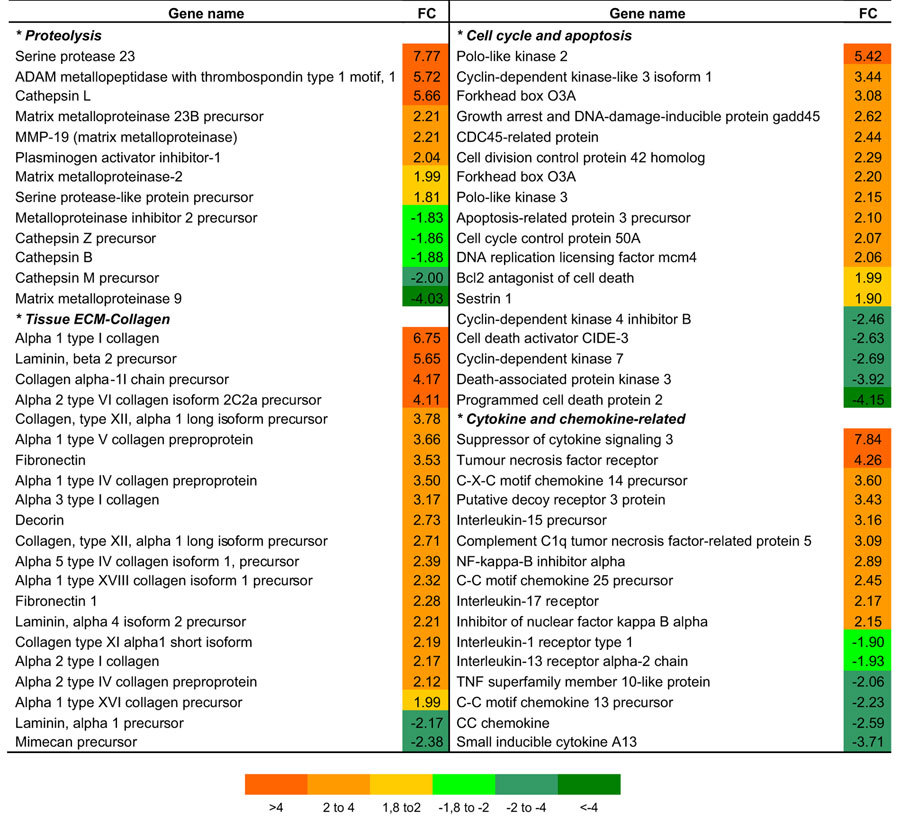
Summary of selected genes expressed in brown trout preovulatory follicles after sLh treatment.

Data shown represent mean fold change (FC; n = 4). Significantly up- and down-regulated genes (*P* < 0.01, Student’s t-test) are highlighted with color scale.
