# Supplementary material for: Luteinizing hormone induces ovulation via tumor necrosis factor α-dependent increases in prostaglandin F2α in a nonmammalian vertebrate
Source: Sci Rep. 2015 Sep 16;5:14210. doi: 10.1038/srep14210 (PMC4570979; doi:10.1038/srep14210)
Supplement: Supplementary Information [file srep14210-s1.pdf]

SUPPLEMENTARY INFORMATION

Luteinizing hormone induces ovulation via tumor necrosis factor α-dependent increases in prostaglandin $F_{2\alpha}$ in a nonmammalian vertebrate

Diego Crespo[a,*], Frederick W. Goetz[b,†] and Josep V. Planas[a]

[a] *Departament de Fisiologia i Immunologia, Facultat de Biologia, Universitat de Barcelona and Institut de Biomedicina de la Universitat de Barcelona (IBUB), Avinguda Diagonal 643, 08028 Barcelona, Spain*

[b] *School of Freshwater Sciences, University of Wisconsin-Milwaukee, 600 E. Greenfield Avenue, WI 53204, USA*

Supplementary Table S1. Enrichment of GO classes and KEGG pathways in the list of DEGs obtained in response to sLh (25 ng/mL).

Functional group / Pathway	Features[1]	*P*-value[2]	Vocabulary
Acute-phase response	12 / 32	0.000	GO
Basement membrane	7 / 81	0.048	GO
Blood coagulation	9 / 95	0.010	GO
Cell adhesion	49 / 622	0.000	GO
Cell differentiation	30 / 535	0.029	GO
Cell surface receptor linked signal transduction	9 / 115	0.043	GO
Chemotaxis	9 / 100	0.015	GO
Complement and coagulation cascades	18 / 115	0.000	KEGG
ECM-receptor interaction	34 / 136	0.000	KEGG
Endopeptidase inhibitor activity	7 / 35	0.000	GO
Extracellular space	39 / 438	0.000	GO
Focal adhesion	49 / 352	0.000	KEGG
Growth factor activity	10 / 104	0.005	GO
Heparin binding	16 / 96	0.000	GO
Inflammatory response	22 / 247	0.000	GO
Insulin-like growth factor binding	9 / 25	0.000	GO
Integrin complex	7 / 45	0.000	GO
Integrin-mediated signaling pathway	16 / 89	0.000	GO
Intermediate filament	15 / 68	0.000	GO
Lipid binding	8 / 47	0.000	GO
Myosin complex	7 / 74	0.027	GO
Natural killer cell mediated cytotoxicity	9 / 113	0.038	KEGG
Platelet activation	8 / 42	0.000	GO
Regulation of actin cytoskeleton	22 / 325	0.007	KEGG
Regulation of cell growth	12 / 108	0.000	GO
Structural constituent of cytoskeleton	22 / 166	0.000	GO
Transforming growth factor beta receptor signaling pathway	6 / 24	0.000	GO
Transmembrane receptor protein tyrosine kinase signaling pathway	17 / 105	0.000	GO

[1] Numbers of genes among DEGs and on the microarray platform. [2] Yate´s corrected chi-square.

Supplementary Table S2. Microarray validation by qPCR. Data are expressed as mean of fold change (FC) ± SEM (n = 4).

ProbeID	Gene name	Microarray	qPCR
Omy#S15300025	Immediate early response gene 2 protein	6.85 ± 2.27	2.15 ± 0.15
Omy#S15296827	Transgelin	4.93 ± 2.35	2.15 ± 0.66
Omy#TC165968	Alpha 1 type IV collagen preproprotein	3.50 ± 1.36	1.53 ± 0.54
Omy#CX247152	Forkhead box O3A	3.08 ± 1.25	1.58 ± 0.25
Omy#CX245477	Testosterone 17-beta-dehydrogenase 3	2.86 ± 0.64	1.58 ± 0.41
Omy#TC151257	Matrix metalloproteinase-2	1.99 ± 1.01	2.35 ± 0.35
Omy#S15244274	Cyclin-dependent kinase 4 inhibitor B	-2.46 ± 1.34	-1.03 ± 1.23
Omy#S22164837	CC chemokine	-2.59 ± 0.91	-2.28 ± 1.51
Omy#S34311675	Transferrin	-3.01 ± 1.57	-1.11 ± 0.69
Omy#S15332001	Matrix metalloproteinase-9	-4.03 ± 0.45	-7.04 ± 1.93
Omy#S34422573	Type I keratin E7	-4.23 ± 1.23	-1.77 ± 0.61
Omy#S22927555	C-type lectin, superfamily member 14 isoform 1	-5.54 ± 2.32	-1.33 ± 1.24

Supplementary Table S3. Sequences of primers used in gene expression analyses by qPCR.

GenBank /ProbeID	Gene name	Primer sequence (5´-3´)	Amplicon size (bp)
AF308735	18S ribosomal RNA (*18s*)	(F) CGGAGGTTCGAAGACGATCA<br>(R) TCGCTAGTTGGCATCGTTTAT	62
DY696186	ADAM metallopeptidase domain 17 (*adam17*)	(F) TGGTGGTCTTCTCGTTGGTCTTC<br>(R) TCCACACAGTGAACCAGGATGC	58
Omy#TC165968	Alpha 1 type IV collagen preproprotein (*col4a1*)	(F) CGAGGACGAGGAGATGTTTACGAA<br>(R) TAAGGGTCACAGAGAGGTCAGGG	134
Omy#S22927555	C-type lectin, superfamily member 14 isoform 1 (*clec14*)	(F) AAGGGGCGACCAACACTCATTAT<br>(R) CTTCTCGGCTTACCAGGCTTCAT	129
Omy#S22164837	CC chemokine (*ccc*)	(F) AGAAGATCACCGTTCCCATCATCG<br>(R) CAGGACTCTTTCCAATGGCTGC	121
Omy#S48436481	Cyclin-dependent kinase 4 inhibitor (*cdkn4b*)	(F) TTACACGATGCCGCCAGGAT<br>(R) TTGTCCCGGCTGTTCGGATC	83
Omy#CX247152	Forkhead box O3A (*foxo3a*)	(F) CTCACAACCACAACCAGGGCTC<br>(R) CTCCCGTTCAGGAAGAGAGACGA	160
Omy#S15300025	Immediate early response gene 2 protein (*ier2*)	(F) AGAGAGCGAGACTTTCACTGGAGC<br>(R) TTTGAGTTAGGGACAGGGGTGGT	109
AF005026	Kallikrein trout #14 (*kt14*)	(F) CCAGGAGGTGGAATTAGGATGAGA<br>(R) GCAGAGCAGCAGTCAGGAACA	167
AB021698	Matrix metalloproteinase-2 (*mmp2*)	(F) CGTCAAACTGGGCGAGATTACA<br>(R) AGGATAGGCTGGTTGGATAGGGA	123
Omy#S15332001	Matrix metalloproteinase-9 (*mmp9*)	(F) CTATGGCACTGTTTCTGGGCTGA<br>(R) GGAGAGACAGAGAATGGCATAGCA	122
AF158374	Prostaglandin-endoperoxide synthase 1 (*ptgs1*)	(F) GAGCCGCCACCAAGGAAACA<br>(R) CAGGCATCATAGGTGGACAAGGG	119
AF158373	Prostaglandin-endoperoxide synthase 2 (*ptgs2*)	(F) TGTCCGATGGTGTCCTTTCAA<br>(R) TGAGAGCAGCTTCAGAACACTGAG	202
Ssa#S47624978	Serine protease 23 (*prss23*)	(F) TGCTGAGGAAGTGAAGTTGACCA<br>(R) GTTGTGTGTTGGCAGGATGTCTC	134
Omy#CX245477	Testosterone 17-beta-dehydrogenase 3 (*hsd17b3*)	(F) GGACAAGACCTACGGCAGCATC<br>(R) CCTATGTCCCCACTCTTTTCTTCA	134
Omy#S34311675	Transferrin (*tf*)	(F) TGTGTCCATCTTTCTGAGCAGCA<br>(R) TTGTGGAATCCTTGAAGAGCGAG	112
Omy#S15296827	Transgelin (*tagln*)	(F) CACACACACTATGACCAACCCAAT<br>(R) GGTTAGTTGCTTCAGTGCCTTCTC	190
U67854	Trout ovulatory protein-2 (*top2*)	(F) GGGATGTGTGCGGAGTTATGCT<br>(R) ACTGTGTCGGTGCAATGCAGTCAT	99
AJ278085	Tumor necrosis factor α (*tnfα*)	(F) AGCATGGAAGACCGTCAA<br>(R) TTCGTTTACAGCCAGGCT	271
209733821	Tumor necrosis factor α-stimulated gene 6 (*tsg6*)	(F) GGCTGAACAAGAGTGAAAGATGGG<br>(R) CTGCTCATCCTGGTACTCCTCTGG	131
Omy#S34422573	Type I keratin E7 (*krt1e7*)	(F) AGGAGTTGGGTCGCATGAAGA<br>(R) TCTCCCGACATCCTCTCCGT	134

F: forward, R: reverse.

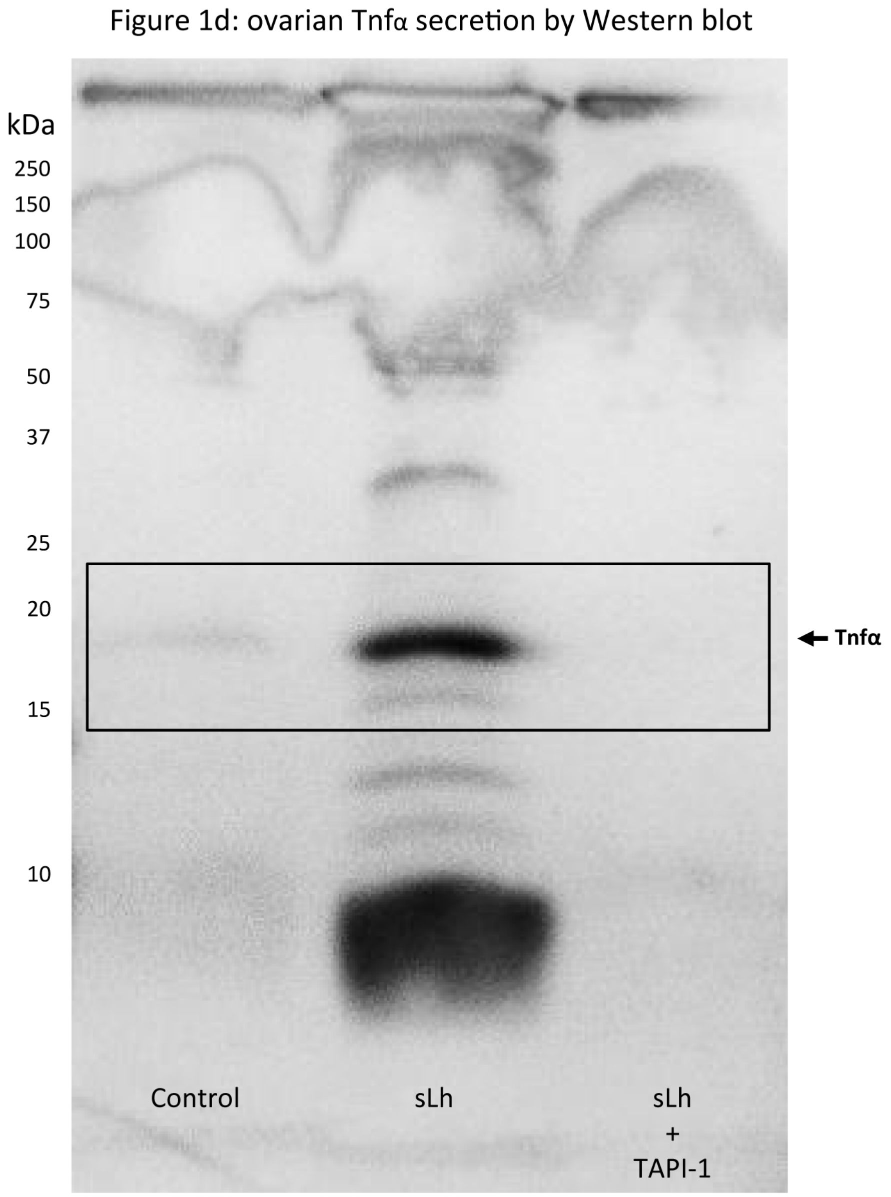

Supplementary Figure S1. Full-length blot image for Figure 1. Box indicates the cropped image shown in Figure 1d.

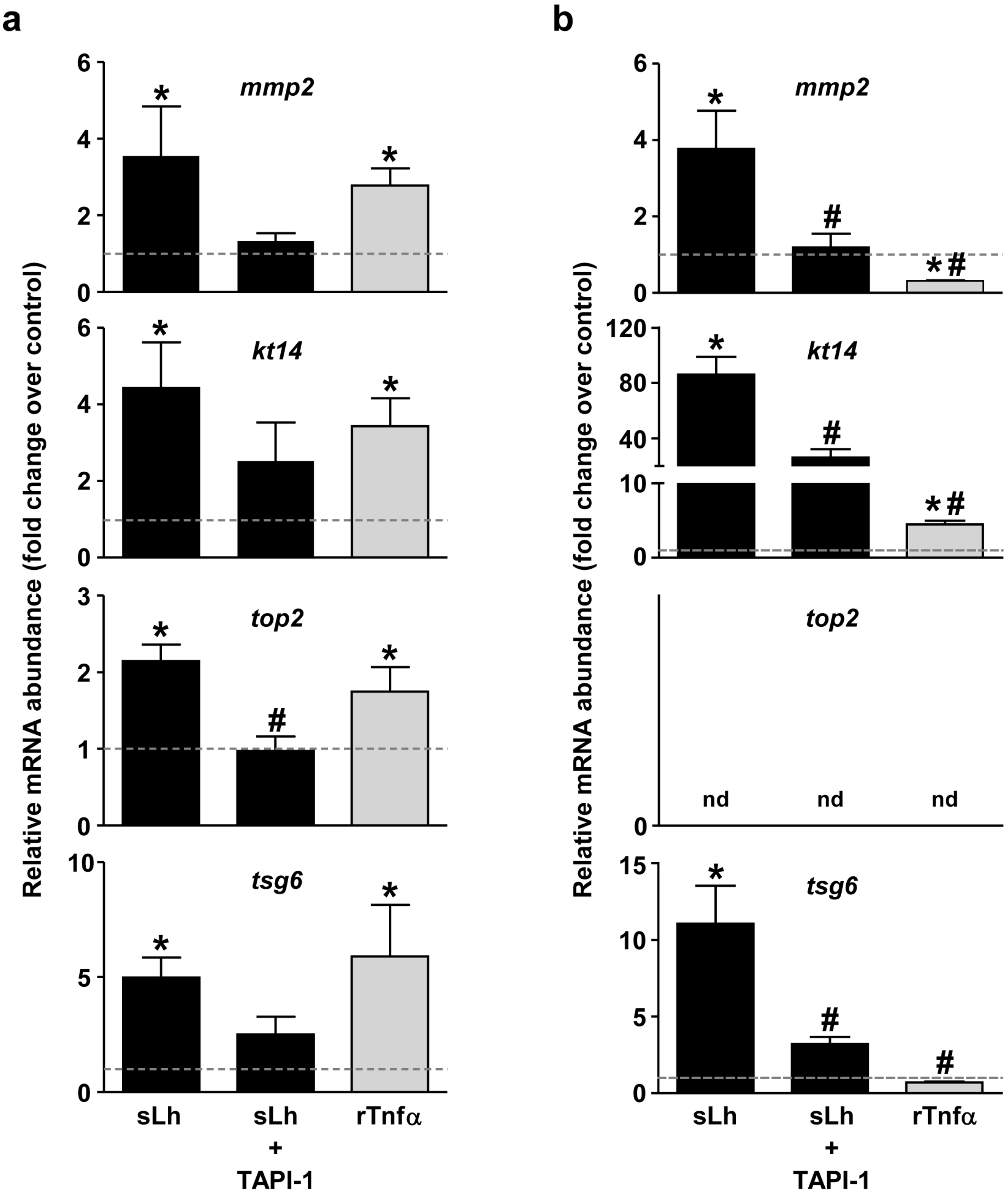

Supplementary Figure S2. Regulation of proteolytic gene expression by Lh and Tnfα in isolated theca (a) and granulosa layers (b) from brown trout preovulatory follicles. Follicles were incubated with sLh (25 ng/mL) in the absence or presence of TAPI-1 (50 μM), or with rTnfα (50 ng/mL). The relative expression of *mmp2*, *kt14*, *top2* and *tsg6* was determined by qPCR and normalized to the abundance of *18s*. The results from ovarian tissue from three separate brown trout females assayed in triplicate are expressed as mean of fold change ± SEM with respect to the control that was set at 1. * $P < 0.05$, with respect to control; # $P < 0.05$, with respect to sLh; nd, no detectable expression.

Figure 2d: Mmp2 activity by gelatin zymography

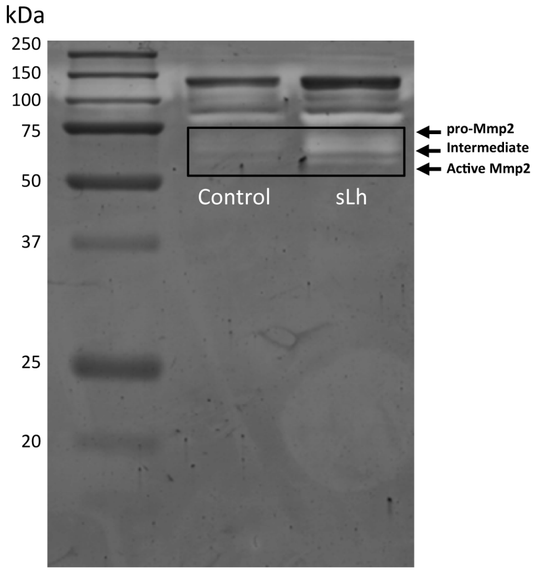

Figure 2e: Mmp2 activity by gelatin zymography

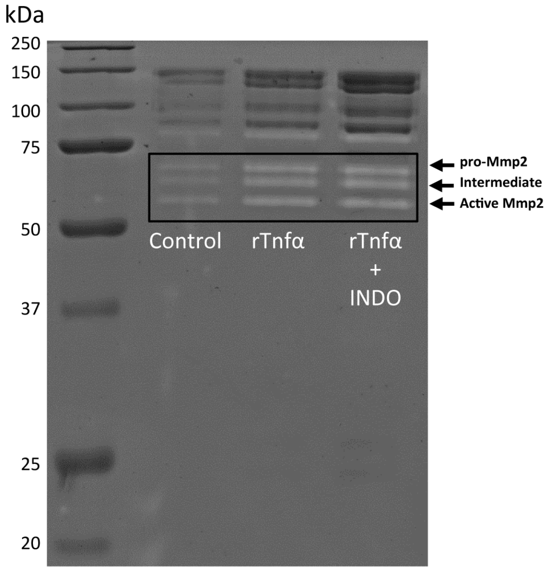

Supplementary Figure S3. Full-length gel images for Figure 2. Boxes indicate the cropped images shown in Figures 2d and 2e.

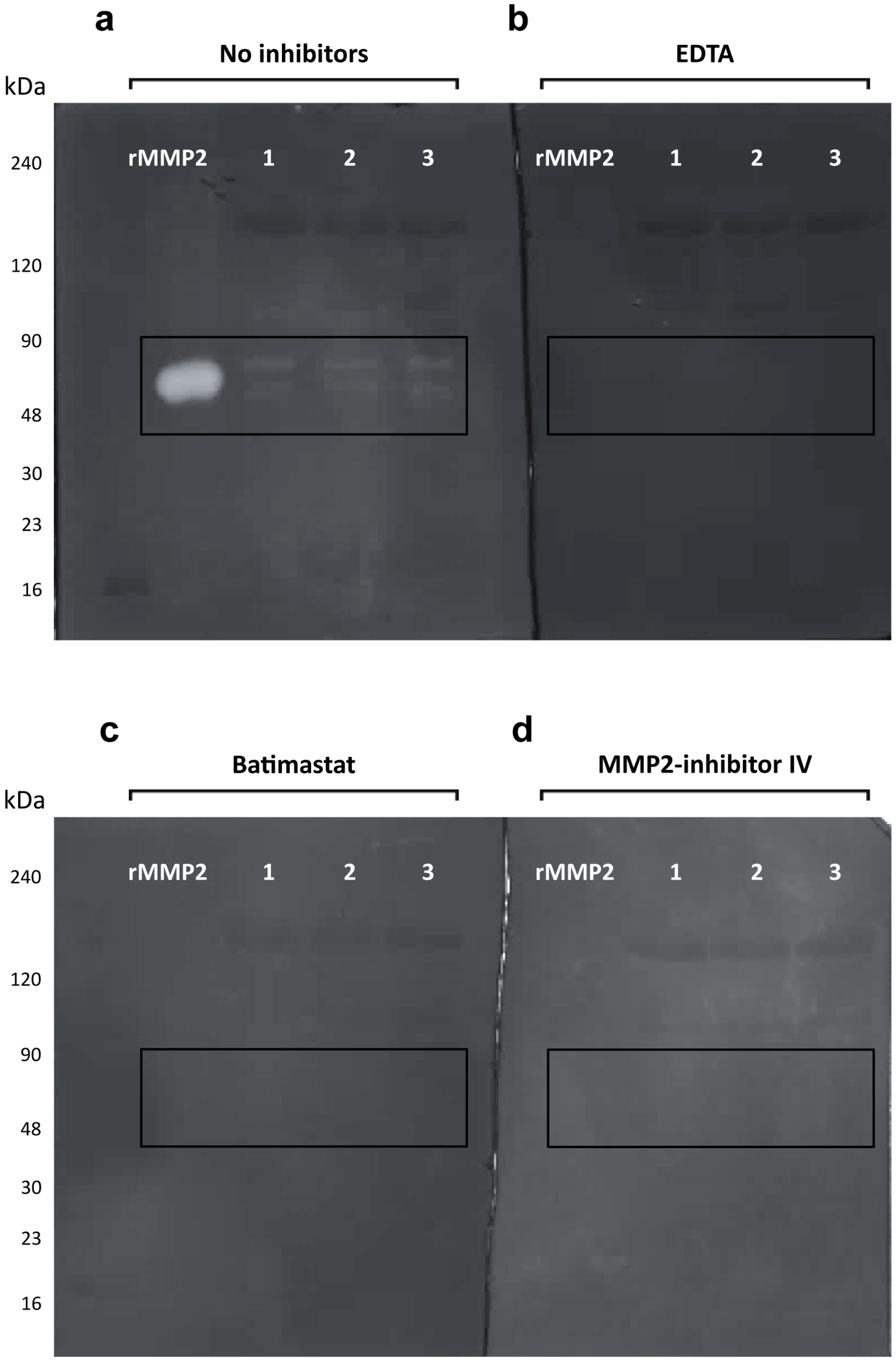

Supplementary Figure S4. Abrogation of gelatinolytic activity by MMP inhibitors. A set of purified follicle homogenates (1, 2 and 3) was run in parallel under the same conditions and gels were subsequently incubated in the absence (**a**) or presence of different MMP inhibitors (broad, **b** and **c**; and MMP2-specific, **d**). Boxes indicate the expected size of MMP2 gelatinolytic activity. Clear bands represent enzymatic digestion of gelatin. rMMP2, recombinant human MMP2.

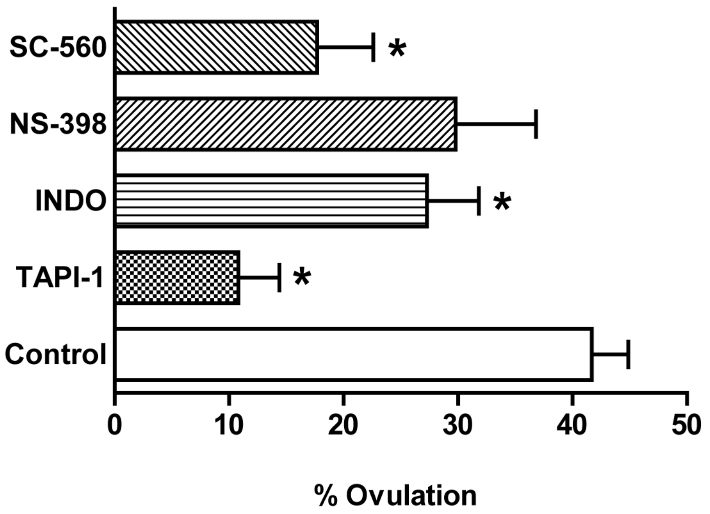

Supplementary Figure S5. Basal effects of various inhibitors on *in vitro* ovulation in brook trout preovulatory follicles. Follicles were incubated for 36 h at 12°C in the presence or absence of TAPI-1 (50 μM), INDO (10 μg/mL), NS-398 (10 μg/mL) and SC-560 (10 μg/mL). Each bar represents the mean ± SEM from four independent experiments, each performed with preovulatory follicles from a different female and assayed in triplicate. * $P < 0.05$, with respect to control.

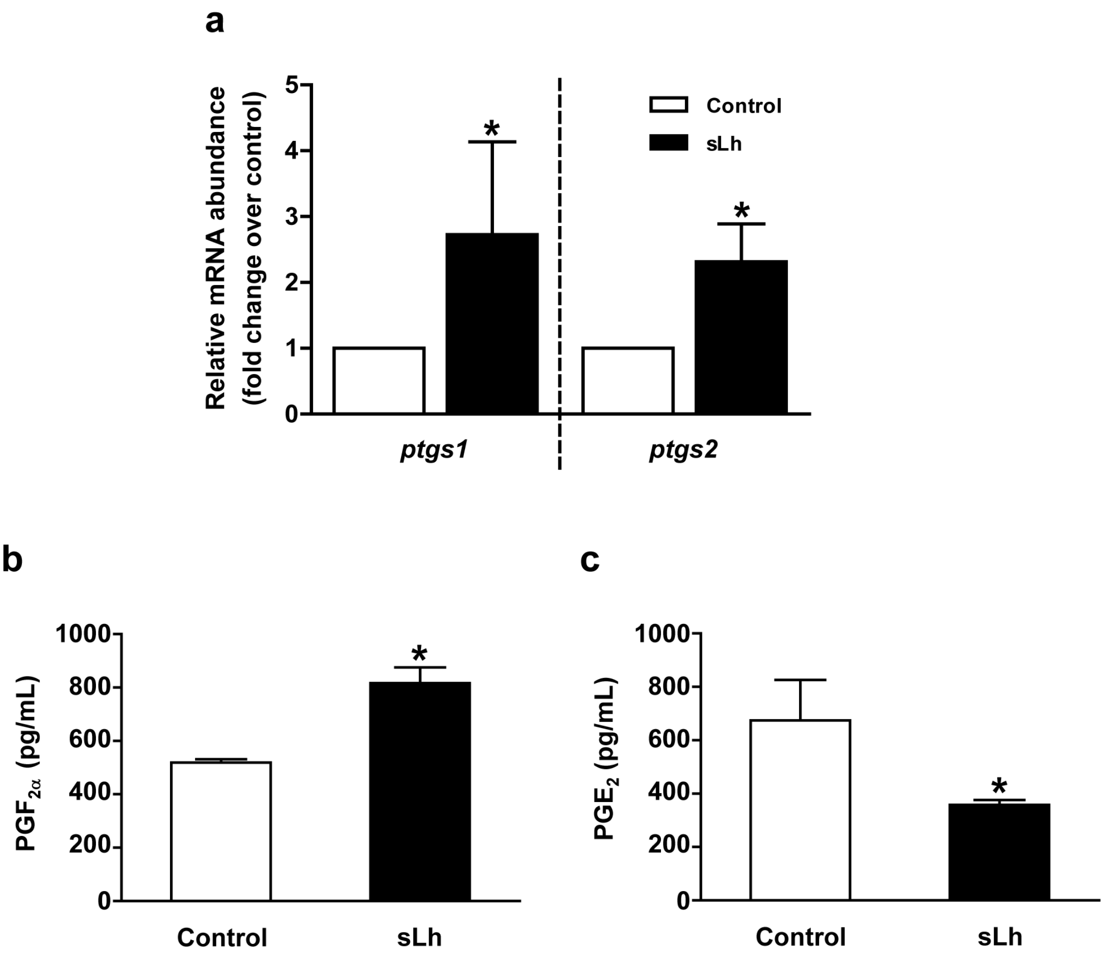

Supplementary Figure S6. Regulation of prostaglandin synthesis by Lh in brook trout preovulatory follicles. (**a**) *In vitro* effects of sLh (25 ng/mL) on the expression of *ptgs1* and *ptgs2* as determined by qPCR and normalized to the abundance of *18s*. The results from ovarian tissue from four separate brown trout females for each group are expressed as mean of fold change ± SEM with respect to the control that was set at 1. * $P < 0.05$, with respect to control. Effects of sLh (25 ng/mL) *in vitro* $PGF_{2\alpha}$ (**b**) and PGE_2 (**c**) production by intact brook trout ovarian follicles. Each bar represents the mean ± SEM of four independent experiments, each with ovarian tissue from a separate female and assayed in triplicate. * $P < 0.05$, with respect to control.

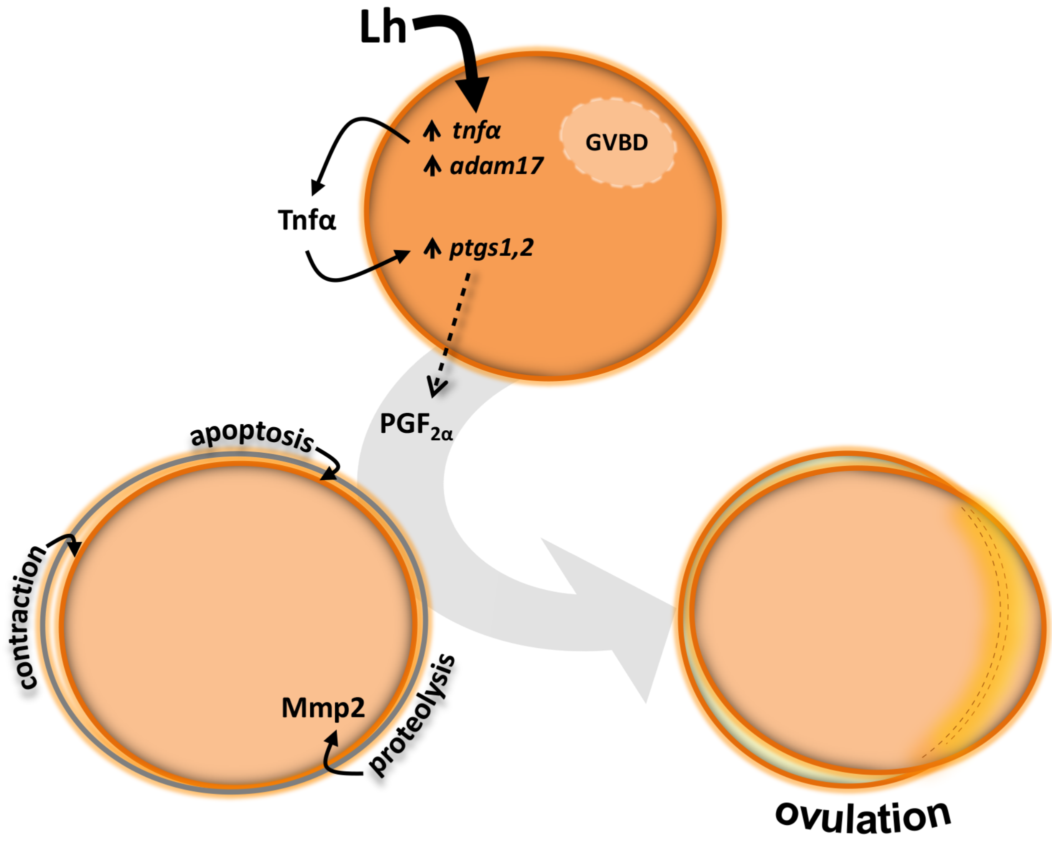

Supplementary Figure S7. Proposed model of the pro-ovulatory actions of Lh in the trout ovary. Evidence from this study strongly supports the notion that Lh orchestrates the complex events leading to ovulation involving the stimulation of proteolysis, apoptosis and contraction of the follicle wall through the stimulation of the local production of $PGF_{2\alpha}$ in a process that is dependent on the ability of Lh to stimulate intraovarian Tnfα production.
